# Studying Gene Expression System Regulation at the Program Level

**DOI:** 10.1371/journal.pone.0061324

**Published:** 2013-04-23

**Authors:** Mark D. Alter

**Affiliations:** Center for Neurobiology and Behavior, Department of Psychiatry, University of Pennsylvania, Philadelphia, Pennsylvania, United States of America; King Faisal Specialist Hospital and Research Centre, Saudi Arabia

## Abstract

Understanding how gene expression systems influence biological outcomes is an important goal for diverse areas of research. Gene expression profiling allows for the simultaneous measurement of expression levels for thousands of genes and the opportunity to use this information to increase biological understanding. Yet, the best way to relate this immense amount of information to biological outcomes is far from clear. Here, a novel approach to gene expression systems research is presented that focuses on understanding gene expression systems at the level of gene expression program regulation. It is suggested that such an approach has important advantages over current techniques and may provide novel insights into how gene expression systems are regulated to shape biological outcomes such as the development of disease or response to treatment.

## Background

**Figure 1:**
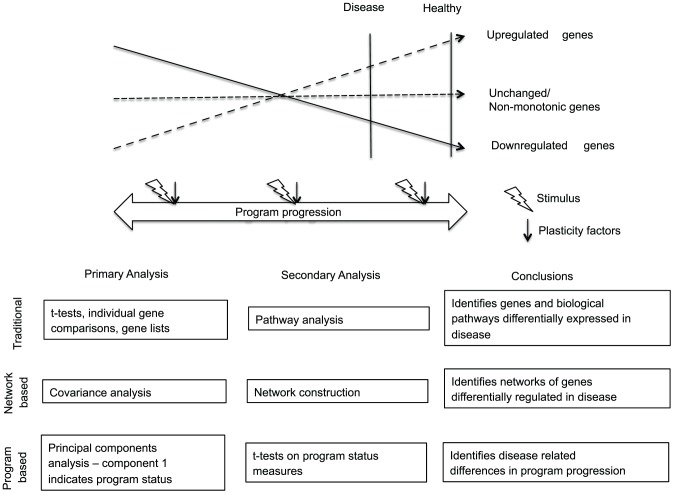
Varied approaches to gene expression profiling data. Schematic compares several approaches to gene expression profiling data. Gene expression levels follow stereotyped patterns as gene expression programs progress. Stimuli (lightening bolts) push programs in one direction or the other. Plasticity factors (arrows) influence how far a program will move in the direction it's pushed. In the example, a gene expression program does not fully progress in disease. Incomplete progression could be related to problems with stimuli or plasticity factors. Under this scenario, gene expression profiling is compared between diseased and healthy individuals. Traditional analysis compares individual genes. Students' t-tests generate lists of genes that are significantly up or downregulated in disease. Lists of genes may be probed with pathway analysis to identify biological pathways that are overrepresented in up- or down-regulated gene lists. A network approach looks at covariance of gene expression levels between diseased and healthy samples. Networks are constructed based on covariance measures with significant covariance representing a connection between genes. The most connected genes are considered to be network hub genes at the top of a hierarchal network. Network analysis identifies gene expression networks differentially regulated in disease. Program-based analysis characterizes transcriptomes in terms of their position along a gene expression program (program status). The first principal component of a principal components analysis measures gene expression program status. Program status measures are compared between diseased and healthy individuals. Program-based approaches identify differences in gene expression program status in disease.

Gene expression programs represent stereotyped (occurring the same way every time) changes in gene expression levels that occur as cells transition from one phenotype to another (figure 1). Expression programs are specific to cell types and support functional changes in cells. Predictable programs arise in the context of complex biological networks of interactions such as positive and negative feedback loops that help systems to self-organize [1]. Stereotyped gene expression programs represent a measurable aspect of self-organizing cellular systems that more broadly encompass dynamic interactions of gene expression levels, protein levels, protein modifications, second messenger systems, and many other biological processes [2], [3], [4], [5], [6].

Within this context, expression programs are classically viewed as windows onto biological processes. For instance, by measuring gene expression changes that occur during differentiation of a neuronal subtype, one might gain insight into factors important for that subtype's unique functional characteristics [7]. More generally, however, gene expression programs may also be viewed as measures of biological time indicating a transcriptome's relative position along a stereotyped gene expression program (program status)(figure 1). Thinking about transcriptomes from a program status perspective may facilitate efforts to understand how gene expression systems are regulated and compliments extensive work by the gene expression research community on the biological details of gene expression programs and how biological networks that support expression programs are constructed [1].

As gene expression systems are orchestrated by expression programs, measuring a transcriptome's position along a gene expression program (program status) may be important to understanding how the state of gene expression systems relate to cell function. A program status-based approach differs from commonly used gene expression research strategies because it treats transcriptomes as single dynamic units constrained by gene expression programs that support functional changes in cells (figure 1). Such an approach is based on the idea that most if not all biologically relevant changes in gene expression levels occur in the context of transcriptome-wide gene expression programs. Under this conceptualization, questions in gene expression systems research can be broadly divided into 2 categories: 1) those that assess details of gene expression programs and the complex networks of interactions that constrain their behavior; and 2) those that seek to understand how gene expression programs are regulated as units.

Each approach has advantages and drawbacks. A detail-oriented approach provides information about what a gene expression program does. For example, the developmental program of fast-spiking interneurons supports changes in ion channels and energy metabolism that underlie changes in firing properties occurring as these cells mature [7]. A program-based approach provides information about how the status of programs relates to higher-level phenomena. For instance, the gene expression program of fast-spiking interneurons was immature in the brains of individuals with autism, bipolar, and schizophrenia [8]. Thus, a detailed approach is important for understanding the cellular consequences of gene expression program regulation, and program status approaches are useful for understanding how regulation of gene expression programs relates to higher-level biological outcomes.

Previously, it was found that gene expression program status could be measured at the transcriptome level by creating a very simple index measure that was the average expression level of all genes in a transcriptome that were up-regulated over the course of a program divided by the average expression level of genes that were down-regulated. This simple index when applied to time course transcriptome profiling data reduced transcriptome data to single measures that tracked closely with developmental time [8]. The index worked well because gene expression changes are primarily monotonic across developmental programs and most stimulus-induced gene expression programs. Thus, a simple averaging approach that divides genes broadly into categories by direction of change will capture the average behavior of thousands of monotonically changing genes in a single measure that tracks with program status. Gene expression levels that do not change expression during a program and those that do not have monotonic temporal profiles will either cancel each other out or will be relatively small in number so as to not affect the overall measure of program status. This approach, however, has the problem that one must know which transcriptomes are at the beginning and end of a program in order to know which genes are up- or down-regulated across a program.

Here we report that principal components analysis (PCA) can similarly reduce transcriptome data to single measures of program status without the need for temporal information. This is important when the order of transcriptomes with respect to a program is unknown. PCA works well for the same reason that an averaging approach works, namely that program related changes in gene expression levels are primarily monotonic. A high level of monotonic gene expression change means inevitably when an expression program generates variability in gene expression levels in a data set, the first principal component will capture the major monotonic data dimension as the first principal component. Therefore, the first principal component will track with the status of the predominant gene expression program accounting for variability in gene expression levels.

Even though the first principal component will capture a dominant monotonic component of gene expression programs, PCA has yet to be explicitly used to measure the status of gene expression programs. This is surprising given that PCA and other data reduction methods have been used extensively since the beginning of the microarray era to analyze gene expression profiling data. In fact, over 1,700 articles are retrieved in PubMed with a search for principal components analysis and gene expression. Despite widespread use, data reduction strategies have primarily been used as classification tools to better understand biological details of gene expression responses [9], [10]. Principal components analysis is often used as a similarity measure to subdivide and classify genes into similarly behaving groups called eigengenes [11], [12]. For instance in a highly cited study of dynamic gene expression patterns in yeast, authors used PCA, also known as singular value decomposition, to show that one eigengene was influenced by over/under expression of a cycle regulator but another eigengene was not affected [12]. Grouping genes into clusters helps to appreciate how genes are co-regulated in a gene expression response and allows one to use other data mining strategies like pathway analysis to probe clusters of genes for enriched biological pathways. In this way, important insights can be gained into what is biologically occurring as a program progresses, but detailed approaches will be less informative when thinking about transcriptomes as dynamic units that follow stereotyped programs.

The goal of the current manuscript is to describe a straightforward PCA-based approach to measuring program status. It will be demonstrated how ordering transcriptomes by the first principal component (PCA1) corresponds to the natural temporal order of known gene expression programs. Examples will be given to show how measuring program status provides insights that may not be readily apparent with traditional or network approaches to gene expression data. Program status-based approaches to gene expression data offer a new way to approach gene expression systems research, which may be ideal for biomarker development and provide ways to develop and screen therapeutic strategies aimed at the targeted manipulation of gene expression program status.

## Methods

### General study design

The current study represented a secondary analysis of previously generated data; therefore, no IACUUC or IRB approval was required.

### Microarray data processing

Microarray data sets were obtained from the Gene Expression Omnibus (GEO - http://www.ncbi.nlm.nih.gov/geo/). When present calls were available (Affymetrix 3′ expression arrays), genes were filtered for those with present calls in >51% of samples. When present calls were not available, genes with average expression levels greater than the median were used. For Affymetrix microarrays, .cel files were downloaded and Robust Multiarray Analysis (RMA) was used for data processing. For other array types, a processed data matrix was downloaded from GEO.

### Principal components analysis (PCA)

Five non-overlapping groups of expression levels from 2000 probe sets/group were used for principal components analysis. Subsets of genes were used to allow PCA to be performed rapidly on a standard desktop computer and to demonstrate that PCA performed on any subset of genes gave nearly identical results for PCA1. This illustrates that PCA1 is detecting a unifying systemic property of datasets. Because information about this property is distributed across the entire transcriptome, it can be measured with non-overlapping groups of genes. Probe set groups were selected based on the order of their probe set ID numbers, i.e. Group 1 was probe sets #1-2000, Group was probe sets #2001-4000, etc. PCA was performed in MATLAB on Z-scored data. The first principal component (PCA1) was used as an index of program status. PCA1 from principal components analysis of each of the 5 groups of probe sets were nearly identical.

### Relationship to program status

The relationship of gene expression levels to program status was calculated as the Pearson r correlation coefficient of PCA1 with expression levels of individual genes in MATLAB with the “corr” function.

### Connectivity analysis

To construct an adjacency matrix cross-correlation of gene expression levels was performed. Cross-correlation scores were assessed for significance based on p<0.001. Genes were considered to be connected if they had a significant (p<0.001) cross-correlation. Connectivity was calculated as the number of connections/total number of genes.

### Statistics

Linear and non-linear regression was performed using Prism (Graphpad, LaJolla, CA). ANOVA with Bonferoni correction was also done in Prism. Cross correlation for replicates of PCA1 was performed in MATLAB with the “corr” function.

## Results

### Arranging transcriptomes by program status with principal components analysis

**Figure 2:**
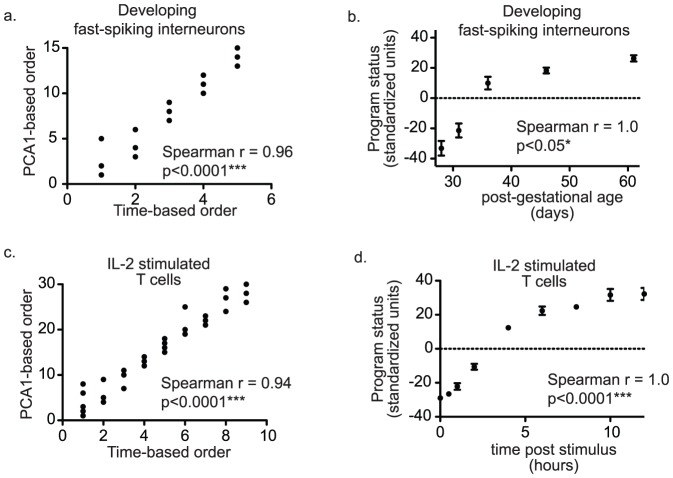
The first principal component (PCA1) measures gene expression program status. Principal components analysis was performed on time course gene expression profiling data. The first principal component score (PCA1) for each transcriptome was used as a measure of program status. Transcriptomes were sorted by PCA1 and their PCA1-based order (y-axis) was compared to their biological time-based order. Panels (2a & 2c) show that PCA1-based and time-based orders are closely related (Spearman r>.94, p<0.0001 for both examples). In panels (2b & 2d), PCA1 is plotted against time to show how program status measures help to visualize gene expression program progression in developing fast-spiking interneurons and in T cells stimulated with interleukin 2 (IL-2).

Many stereotyped gene expression programs involve primarily monotonic changes in gene expression levels. When monotonic program-related changes in gene expression levels are the major type of expression change during program progression, this pattern will be recognized as the first principal component (PCA1) in a principal components analysis. To demonstrate this, principal components analysis was performed on transcriptomes from a developmental time course experiment in parvalbumin-positive fast-spiking interneurons (GSE17806) and from a biological time course following interleukin 2 (IL2)-stimulation of T lymphocytes (GSE6085). The PCA1-based order was then compared to the known biological temporal order of microarray experiments. The biological order based on developmental age or time post IL2 stimulation was significantly related to the order based on the first principal component score (PCA1) for each array (figure 2a & c). A close relationship between PCA1-based and time-based orders suggested that PCA1 would make an excellent measure of gene expression program status. Indeed, in both experiments PCA1 increased over time with an initially steep increase as the program rapidly progressed followed by a slowing of program progression (figure 2b & d). Thus, plotting PCA1 against time provided a useful visual representation and quantification of gene expression program progression.

### Program status correlates with gene connectivity

A well-established feature of gene expression systems is a hierarchal data structure. In this type of system a limited number of genes at the top of a hierarchy control genes below them that in turn influence other genes and so on. Such a structure can be appreciated with network analysis where gene expression networks are reverse engineered using connectivity matrices [1], [13], [14], [15], [16]. In this sense, genes are defined as connected if their gene expression levels move together. Connectivity is quantified as the number of genes with significant covariance divided by the total number of genes. Thus, genes at the top of a hierarchy influence many genes below them and will have high connectivity, whereas, genes lower in the hierarchy will be related to fewer genes and have lower connectivity.

**Figure 3:**
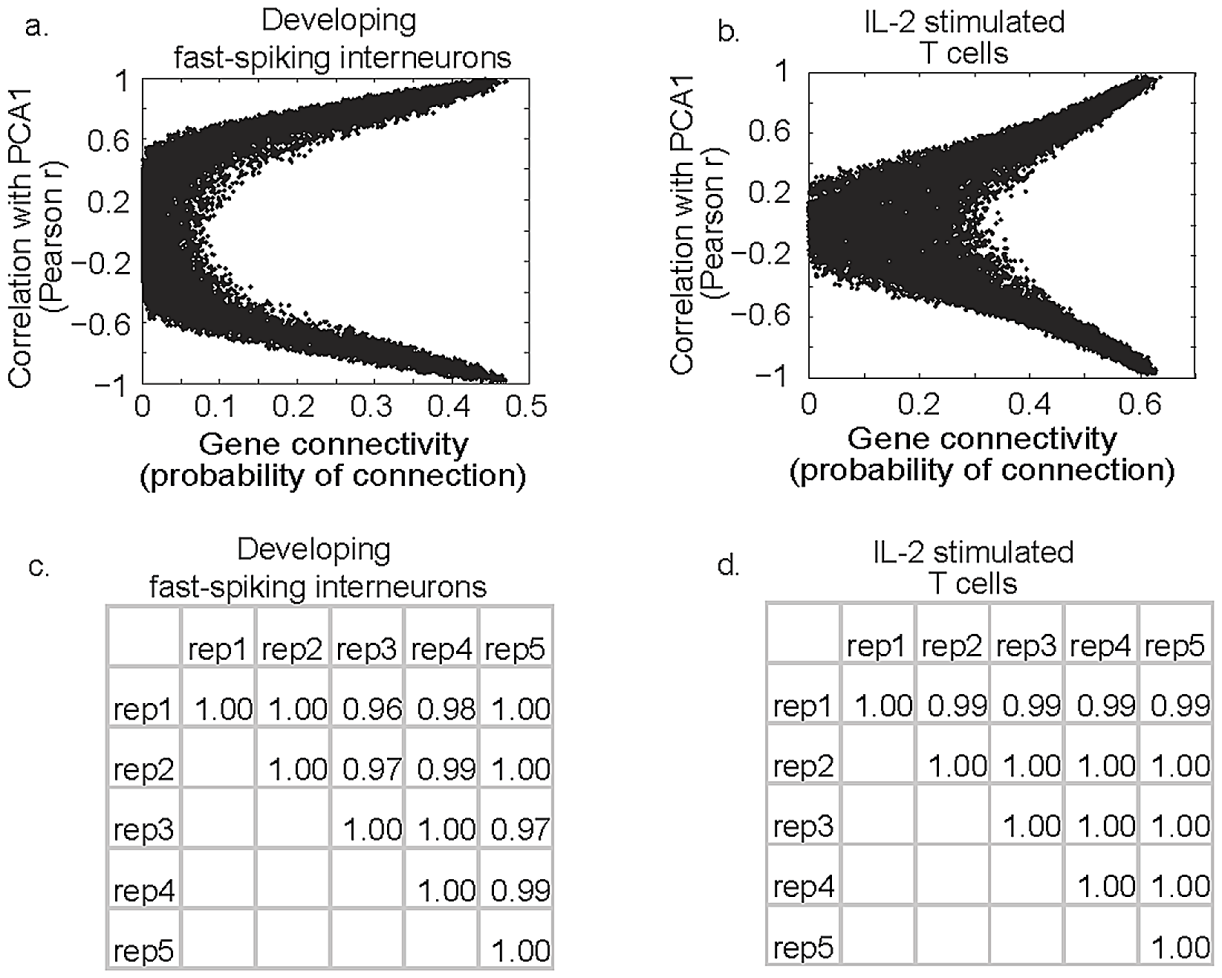
Connectivity of genes relates to how well genes follow gene expression programs. Gene connectivity was calculated as the number of genes that had significant covariance with a gene over the total number of genes. Connectivity (x-axis) was compared to a gene's Pearson r value for its correlation with program status (PCA1). Panels (3a & 3b) demonstrate that the most connected genes have the highest absolute Pearson r values indicating that highly connected genes follow more closely with programs than less connected genes. Panels (3c & 3d) are cross-correlation tables for PCA1 calculated using independent groups of 2000 probesets from the same transcriptomes. Tables show that PCA1 values are nearly identical even when completely different genes from the same transcriptomes are used for principal components analysis.

This well-established property of gene expression networks was used to demonstrate in another way that PCA1 was a measure of gene expression program status. Genes at the top of the hierarchy, termed systemic hub genes, influence the largest number of genes and, therefore, their expression levels were predicted to be most closely associated with program status. To assess how well a gene's expression level followed with a program, gene expression levels were correlated with program status (PCA1). A Pearson r correlation coefficient was calculated for each gene based on the linear relationship of its expression level to PCA1. This was used as a measure of how well a gene followed a program and was compared to a gene's connectivity. As would be expected in a hierarchal biological system, a gene's connectivity was related to how closely the gene followed a gene expression program (figure 3a–b). This was true regardless of whether a gene increased or decreased its expression level during the course of the program.

A hierarchal data structure in the context of a stereotyped and primarily monotonic gene expression program also meant that PCA1 obtained with one subset of a transcriptome should agree with results from another subset even when subsets did not share any of the same genes. This is true because each group of genes should contain redundant information about the status of a shared gene expression program. To demonstrate this, PCA was performed on 5 independent subsets, each consisting of 2000 probe sets. PCA1 scores from these independent analyses were then compared and demonstrated near perfect correlation between replicates (figure 3c–d).

### Euclidean distance between transcriptomes further validates PCA1 as a measure of program status

**Figure 4:**
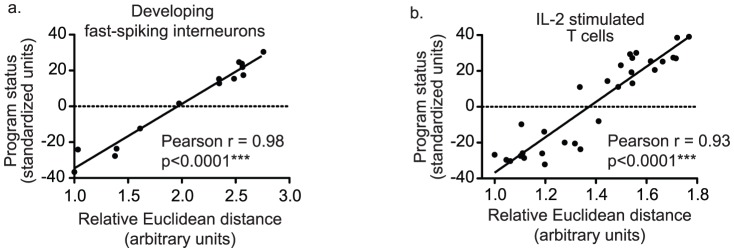
Program progression is linearly correlated with dissimilarity measures. Euclidean distance (x-axis) for each transcriptome was calculated from an index transcriptome with the lowest value for PCA1. Euclidean distance was used as a measure of absolute dissimilarity. Distance was plotted against program status (y-axis) to demonstrate a linear relationship between program status (increasing PCA1) and dissimilarity (Euclidean distance) for gene expression programs in developing fast-spiking interneurons (4a) and IL-2 stimulated T cells (4b).

As transcriptomes progress along gene expression programs, genes expression levels do not change randomly, rather changes in gene expression levels are constrained to stereotyped and primarily monotonic changes in expression levels. Therefore, dissimilarity between transcriptomes should increase linearly with program progression. This predicted system level property was used to further validate PCA1 as a measure of program status. Euclidean distance was used as a dissimilarity measure between individual transcriptomes and a reference transcriptome at the beginning of a program, which was identified as the transcriptome with the lowest first principal component score. As would be expected in the case of a stereotyped gene expression program, dissimilarity between transcriptomes was highly correlated with program status measures (figure 4a–b).

### Program status measures reveal gene expression system modulation at the program level

**Figure 5:**
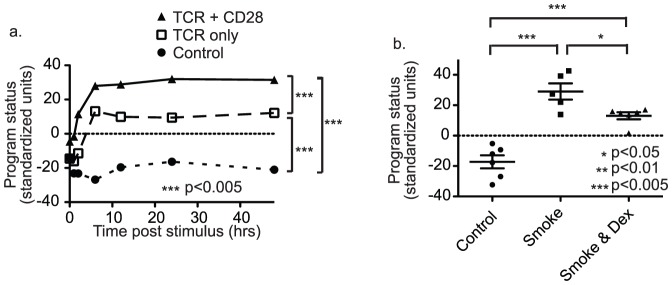
Measuring gene expression system modulation at the program level. Panel (5a) shows a time course of program status measures after stimulation of the T cell receptor (TCR) alone or in combination with the co-stimulatory molecule (CD28). Results are compared to time-matched non-stimulated controls. Plot shows that co-stimulation with CD28 increases gene expression program progression resulting in a higher plateau in terms of program status. There was a significant difference in plateau program status measures (6–48 hour post-stimulation). Significance was calculated using repeated measures one-way ANOVA with Bonferoni correction for multiple testing. Panel (5b) shows a comparison of gene expression program status in tracheal explant cultures 24 hours after stimulation with smoke or smoke with dexamethasone pretreatment [18]. Results were compared to time matched untreated explants. Plot shows that pretreatment with dexamethasone decreases gene expression program progression. There was a significant difference between program status measures in smoke and smoke plus dexamethasone conditions. Significance was calculated using one-way ANOVA with Bonferoni correction for multiple testing.

An ability to explicitly measure and quantify gene expression program status made it possible to examine modulation of gene expression systems at the program level. To demonstrate the advantages of a program status-based approach, a gene expression study comparing T cell stimulation with T cell receptor alone or in combination with a co-stimulatory molecule, CD28, was examined [17] (GSE3630). In this study it was reported that CD28 primarily increased gene expression changes in the same direction as those that occurred with T cell receptor alone [17]. Monotonic changes in gene expression levels were consistent with the possibility that CD28 increased progression of a T cell receptor induced gene expression program. Indeed, examination of data from the same transcriptome profiling experiment revealed that a T cell receptor-induced gene expression program progressed further with CD28 co-stimulation (figure 5a). Similarly, dexamethasone, a glucocorticoid that inhibits multiple cellular responses, attenuated the progression of a smoke-induced gene expression program in tracheal explant cells (figure 5b) (GSE15563). Together, these studies provided proof-of-principle examples of the ability to examine gene expression profiling data from a program status perspective. Such examples indicate that gene expression program status is a biological target of gene expression system modulation.

To confirm gene expression program modulation in the above experiments, studies were examined from a traditional gene expression analysis perspective. If programs were truly modulated, then a traditional analysis would reveal predictable patterns. Specifically, when significant gene expression changes occurred in the context of a modulated gene expression program, gene expression changes would occur in the same direction in both groups and consistently less in one group relative to the other. In both studies, a large proportion of gene expression levels significantly changed by at least 1.1-fold in one treatment group (48% in the T cell study and 39% in the smoke study). Of these, 90% (chi-squared = 4,638; p<0.0001) and 98% (chi-squared = 8,488; p<0.0001) respectively had significant changes in the same direction across modulated and non-modulated responses. Of genes that were significantly changed in at least one treatment group and in the same direction in both groups, 26% and 17% respectively were significantly different between the modulated and non-modulated groups. In the case of the T cell study, 90% (chi-squared = 1,093; p<0.0001) of modulated genes changed more in the presence of CD28, whereas, in the smoke study 99% (chi-squared = 1,483; p<0.0001) of modulated genes changed less in the presence of dexamethasone. Thus, in both studies, results demonstrated a consistent direction of gene expression modulation supporting regulation at the gene expression program level.

### Program status measures are not affected by the identity of genes used for PCA

If the first principal component is truly measuring the status of unifying stereotyped gene expression programs, then information about the status of gene expression programs should be widely distributed across transcriptomes. To test this hypothesis, PCA was performed on completely independent groups of genes and PCA1 scores were compared across independent analysis. Indeed, the measurement of PCA1 was not sensitive to the group of genes used. Five independent groups of 2000 probe sets gave nearly identical values for PCA1 in all experiments (table 1). Thus, PCA1 was an extremely robust measure that captured a systemic property of the dataset that was not dependent on gene expression levels used in the analysis.

**Table 1:** First principal component scores are not sensitive to the identity of gene expression levels used for PCA.

PV maturation							IL2 T cell timecourse						
		rep1	rep2	rep3	rep4	rep5			rep1	rep2	rep3	rep4	rep5
		PCA1	PCA1	PCA1	PCA1	PCA1			PCA1	PCA1	PCA1	PCA1	PCA1
rep1	PCA1	1.00	1.00	1.00	1.00	0.99	rep1	PCA1	1.00	0.99	0.99	0.99	0.99
rep2	PCA1	1.00	1.00	1.00	1.00	0.99	rep2	PCA1	0.99	1.00	1.00	1.00	1.00
rep3	PCA1	1.00	1.00	1.00	1.00	0.99	rep3	PCA1	0.99	1.00	1.00	1.00	1.00
rep4	PCA1	1.00	1.00	1.00	1.00	0.99	rep4	PCA1	0.99	1.00	1.00	1.00	1.00
rep5	PCA1	0.99	0.99	0.99	0.99	1.00	rep5	PCA1	0.99	1.00	1.00	1.00	1.00

T Cell costimulation							Dexamethasone and smoke						
		rep1	rep2	rep3	rep4	rep5			rep1	rep2	rep3	rep4	rep5
		PCA1	PCA1	PCA1	PCA1	PCA1			PCA1	PCA1	PCA1	PCA1	PCA1
rep1	PCA1	1.00	1.00	1.00	0.99	0.99	rep1	PCA1	1.00	1.00	0.98	1.00	0.99
rep2	PCA1	1.00	1.00	0.99	0.99	0.99	rep2	PCA1	1.00	1.00	0.97	0.99	0.99
rep3	PCA1	1.00	0.99	1.00	0.99	1.00	rep3	PCA1	0.98	0.97	1.00	0.98	0.94
rep4	PCA1	0.99	0.99	0.99	1.00	0.99	rep4	PCA1	1.00	0.99	0.98	1.00	0.99
rep5	PCA1	0.99	0.99	1.00	0.99	1.00	rep5	PCA1	0.99	0.99	0.94	0.99	1.00

For each dataset, PCA was performed on 5 independent groups of 2000 genes/group. The first principal component score (PCA1) for individual transcriptomes were cross-correlated across independent analyses. In all cases, results were nearly identical (Pearson r>0.94) across analyses performed with non-overlapping groups of genes.

## Discussion

Transcriptome profiling data from multiple publically available datasets were analyzed with principal components analysis (PCA) to demonstrate that the first principal component (PCA1) represents a useful measure of gene expression program status. This was demonstrated in a number of ways. It was shown using time course time course data that the temporal order of transcriptomes was highly correlated with the order based on the program status measure, PCA1. It was also demonstrated that PCA1 captured the behavior of a hierarchal gene expression system. Highly connected genes followed most closely with program status. Importantly, measuring program status could also be used to understand modulation of gene expression systems. For instance, modulation of T cell activation with CD28 was shown to increase progression of the same program induced by the T cell receptor alone. Conversely, dexamethasone decreased the progression of a stereotyped program induced by smoke.

Though PCA has been widely used to analyze gene expression data since the beginning of the microarray era, a relationship between PCA1 and gene expression program status has not been previously reported. A recognition and understanding that the first principal component measures the status of most gene expression programs is not trivial. An ability to explicitly measure program status is of critical importance to asking questions about how gene expression systems are regulated. For instance, in the above examples, program status measures demonstrated that functional T cell modulation [17] was closely related to modulation of gene expression program status. Similarly, the induction of cellular plasticity in response to smoke was attenuated by the steroid, dexamethasone [18], and this was related to a similar attenuation of a smoke-induced gene expression program. Thus, program status measures provided a straightforward interpretation of variability in cellular plasticity as closely related to variability in the progression of stereotyped gene expression programs.

Importantly program status measures were highly robust for all datasets examined. Program status measures obtained using five totally independent groups of 2,000 probe sets gave nearly identical results. This is important, because robust and biologically meaningful measures of gene expression system regulation have been elusive. Robust measures are crucial to the potential usefulness of gene expression data in generating reliable and predictive biomarkers.

Program status-based approaches have important differences from current approaches, even systems-based approaches, which treat transcriptomes as thousands of interacting parts and place emphasis on understanding what these parts do and how they interact (figure 1). Such detail-oriented approaches are important and informative and increase understanding of how cells do what they do, but detail-oriented approaches also have drawbacks that might lead researchers to miss the bigger picture of how transcriptomes are regulated to influence cell function. For instance, a tremendous amount of effort has been spent on studying the immense complexity of chromatin level changes that occur around individual genes when their gene expression levels change. Results of this research have been fascinating and provide a glimpse into just how complex is the conceptually simple task of turning a gene on or off [19]. Yet, delving into this complexity can limit research questions to this detailed level. For instance, based on detailed findings one might be tempted to ask questions such as how do chromatin modifications at gene X differ in disease Y? Inevitably, differences can be found that then generate attempts to find ways to exploit these differences to benefit treatment and so on.

The inherent problem with this approach is that cells and disease states may not operate on this detail-oriented level. For instance, global non-specific changes in chromatin modifications are found in response to a great number of stimuli including treatments [20], disease [21], and environment [22]. These global changes are generally treated as non-specific noise. Yet, from a program status perspective non-specific global changes in epigenetic factors may be key to understanding how gene expression systems are modulated. If one thinks about transcriptomes as dynamic units that follow stereotyped cell-type specific programs, then a logical question becomes: What makes a transcriptome move along its program? And, might global non-specific changes in chromatin state facilitate transcriptome movement and by extension cellular plasticity?

Understanding that gene expression systems are modulated at the level of program status could have substantial impact on how gene expression systems are studied and on attempts to develop gene expression system-based therapeutics. A research approach guided by program status-based principles would look very different than current gene expression research strategies. A program status-based approach would aim to determine what cell-type specific gene expression programs have status differences in disease and where these differences are found. Using such an approach, it was found the gene expression program of fast-spiking interneurons was immature in the cortex of patients with autism, schizophrenia, and bipolar disorder [23].

Next, a program status-based approach could examine the pattern of transcriptome changes with successful treatments for disease to see whether program status changes were associated with individual differences in treatment response or resistance. Mechanisms for treatment associated program status changes could be evaluated from the perspective of potential plasticity factors such as global levels of histone acetylation, and when possible such plasticity factors might be modulated via alternative mechanisms to test for a causal relationship to program status modulation.

In addition to plasticity-based approaches, modulation of program status could be explored from a stimulus-based perspective with research aimed at finding stimuli that push gene expression programs in hypothesized therapeutically beneficial directions. Such studies could be informed by studying endogenous regulation of the same programs during development or in response to environmental variables. Studies of program status regulation could be done in animal models of disease or even in cell culture systems in order to inform general principals of regulation. Importantly, these research approaches would avoid time-consuming efforts to delve deeper into the specifics of individual gene expression changes that occur during the progression of cell type specific gene expression programs. By focusing research on easily measurable and potentially targetable aspects of gene expression systems, program-based approaches could accelerate progress and direct research toward actionable findings.
